# Detection of Superoxide Alterations Induced by 5-Fluorouracil on HeLa Cells with a Cell-Based Biosensor

**DOI:** 10.3390/bios9040126

**Published:** 2019-10-16

**Authors:** Sophia Mavrikou, Vasileios Tsekouras, Maria-Argyro Karageorgou, Georgia Moschopoulou, Spyridon Kintzios

**Affiliations:** 1 Faculty of Applied Biology and Biotechnology, Department of Biotechnology, Agricultural University of Athens, Iera Odos 75, 11855 Athens, Greece; tsekouras@aua.gr (V.T.); geo_mos@aua.gr (G.M.); 2 Institute of Nuclear & Radiological Sciences & Technology, Energy & Safety, National Center for Scientific Research “Demokritos”, Aghia Paraskevi, 15310 Athens, Greece; kmargo@phys.uoa.gr; 3 Faculty of Physics, Department of Solid State Physics, NKUA, 15784 Athens, Greece

**Keywords:** anticancer therapeutic strategies, apoptosis, bioelectric, biosensor, 5-fluorouracil, HeLa cell line, superoxide

## Abstract

Background: In vitro cell culture monitoring can be used as an indicator of cellular oxidative stress for the assessment of different chemotherapy agents. Methods: A cell-based bioelectric biosensor was used to detect alterations in superoxide levels in the culture medium of HeLa cervical cancer cells after treatment with the chemotherapeutic agent 5-fluorouracil (5-FU). The cytotoxic effects of 5-fluorouracil on HeLa cells were assessed by the MTT proliferation assay, whereas oxidative damage and induction of apoptosis were measured fluorometrically by the mitochondria-targeted MitoSOX™ Red and caspase-3 activation assays, respectively. Results: The results of this study indicate that 5-FU differentially affects superoxide production and caspase-3 activation when applied in cytotoxic concentrations against HeLa cells, while superoxide accumulation is in accordance with mitochondrial superoxide levels. Our findings suggest that changes in superoxide concentration could be detected with the biosensor in a non-invasive and rapid manner, thus allowing a reliable estimation of oxidative damage due to cell apoptosis. Conclusions: These findings may be useful for facilitating future high throughput screening of different chemotherapeutic drugs with a cytotoxic principle based on free radical production.

## 1. Introduction

Gynecological cancers are life-endangering malignancies of the female reproductive system, discriminated as cervical, ovarian, uterine, vaginal, vulvar, and fallopian tube cancer after the affected anatomical organ. These diseases are estimated to cause approximately 110,000 new cases and 33,000 deaths in the United States alone on an annual basis [1].

The successful treatment of cancers requires a combination of invasive and non-invasive processes such as surgery, chemotherapy and radiation, considering efficient treatment and patient’s quality of life [2]. Chemotherapy is a major constituent of the multidisciplinary cancer therapy, evolved throughout the years from a single active compound remedy to a multifactor approach, applying combined regimens towards personalized medicine. One of the most frequently used chemotherapeutic agents in cancer therapy is 5-fluorouracil (5-FU), an analogue of uracil [3,4]. The anticancer activity of 5-FU originates from the inhibition of thymidylate synthase (TS) activity during the S phase of the cell cycle and its incorporation into RNA and DNA of tumor cells [5,6]. Furthermore, 5-FU promotes cell death by generating mitochondrial ROS in the p53-dependent pathway [7,8,9] and by inducing apoptosis through the activation of a cascade of caspases 1, 3 and 8 [10].

Cancer cells have the ability to prevent programmed cell death by disorganizing tissue homeostasis, the balance between cell proliferation and cell death [11]. Therefore, a fundamental target of conventional chemotherapy is the activation of endocellular signaling mechanisms involved in cell death pathways, in particular, those mediating apoptosis. 5-fluorouracil is an effective pharmaceutical agent reported to initiate apoptotic processes against a number of malignancies such as colorectal [12], oral [13], breast [14], head and neck [15], gastric [16] and cervical carcinomas [17,18]. A crucial element of apoptotic cell death is caspase-3, a cysteine protease that catalyzes a number of key endocellular proteins [19]. Human caspases are a group of eleven endoproteases, caspases 1–10 and caspase 14, controlling cell regulatory networks of inflammation and cell death [20]. The executioner caspase-3 is activated from initiator caspases, the outcome of apoptotic cell signaling driven from either mitochondrial cytochrome c release or cell death receptor activation [21]. The anticancer efficacy of 5-FU, when administered alone or in combination with other agents, is associated with caspase-3 function as reported in several in vivo and in vitro studies regarding colorectal [22], gastric [23], pancreatic [24] and gynecological malignancies [25,26].

Cell culture monitoring can be used as indicator for the response to different chemotherapy options. The use of biosensors, in particular bioelectric and electrochemical sensors in the analysis of antineoplastic drugs has increased in importance over the last years [27,28]. In this context, a critical marker for monitoring cancer cells differentiation within a cell population is the superoxide anion. This molecule is mainly a by-product of the oxidative phosphorylation of the mitochondria electron transport chain. Initially, it is released to the mitochondrial matrix, where it is converted immediately to hydrogen peroxide. Mitochondrial hydrogen peroxide can then diffuse to both the cytosol and the nucleus and either interact with other free radical species, modulate signaling cascades or cause cellular damage. Together with other free radical species, superoxide has been found to mediate the development and/or survival of cancer cells and tumors, both in vivo and in vitro [29,30,31]. This property of superoxide has led researchers to propose the regulation of cellular redox status as a novel, critical and highly efficient cancer therapeutic strategy [32,33], utilizing superoxide dismutase along with other antioxidant systems [34,35].

Chemotherapy is associated with oxidative stress alterations that affect vital cellular processes such as cell cycle progression and drug-induced apoptosis [36,37,38]. The scope of this study is to investigate whether it is possible to employ a biosensor-based approach to detect in a non-invasive way the superoxide levels generated by cervical cancer cells after exposure to the anticancer agent 5-fluorouracil. For this purpose, different 5-FU concentrations were tested towards the HeLa cervical cancer line for 24 and 48 h. After the determination of 5-FU’s cytotoxic activity cellular stress markers such as mitochondrial superoxide and caspase-3 levels were determined. Superoxide levels were determined in parallel in the culture medium with an advanced cell-based bioelectric biosensor, an approach which has been previously applied to monitoring superoxide levels in cultures of differentiating neuronal cells [39]. In this way, we demonstrate that it is possible to access in a high throughput, non-invasive way the in vitro efficacy of target anticancer compounds with a cytotoxic principle based on free radical production.

## 2. Materials and Methods

### 2.1. Cell Line and Culture Conditions

HeLa (ATCC^®^ CCL-2™) and Vero cell lines were originally purchased from the American Type Culture Collection (ATCC) (Manassas, VA, USA). The cells were cultured in Dulbecco’s Modified Eagle Medium (BiochromGmbh, Berlin, Germany) supplemented with 10% fetal bovine serum (Thermo Fisher Scientific, Waltham, MA, USA), 2 mM L-glutamine, 0.5 mM sodium pyruvate and 1% penicillin/streptomycin, all procured from Biowest (Biowest, Nuaillé, France). Cells were incubated in a 5% CO_2_ incubator (HF90Air jacketed CO_2_ Incubator, Heal Force Bio-Meditech Holdings Limited, Shanghai, China) at 37 °C for proliferation.

### 2.2. MTT Cell Proliferation Assay

Cells were plated in transparent flat-bottom 96-well plates (SPL Life Sciences Co Ltd., Naechon-Myeon, Korea) at two different population densities according to two incubation time intervals (24 and 48 h): 10^3^ and 8 × 10^3^ cells per well, both supplemented with 100 μL of culture medium. The next day the medium was replaced with 200 μL of medium supplemented with 1% FBS that contained the different 5-fluorouracil (5-FU) concentrations. Cells not treated with 5-FU were considered as control (0). The cytotoxic agent 5-FU was initially diluted in dimethyl-sulfoxide. After 24 and 48 h incubation 0.5 mg/mL 3-(4,5-dimethylthiazol-2-yl)-2,5-diphenyltetrazolium bromide (MTT) (Duchefa Biochemie, Haarlem, the Netherlands) was added in each well. After 3 h in culture, the MTT-containing medium was removed and cells were solubilized with 200 μL dimethyl sulfoxide (DMSO). Cell viability was determined by measuring the absorbance at 560 nm wavelength, using a PowerWave 240 microplate photometer (Biotek, Winooski, VT, USA). The results were expressed as the percentage of absorbance values compared to control and were assessed to determine the changes in viability.

### 2.3. Measurement of Mitochondrial Superoxide Production

Mitochondria-targeted MitoSOX™ Red fluorogenic dye (Thermo Fisher Scientific, Rockford, IL, USA) was used to measure mitochondrial superoxide accumulation according to the manufacturer’s instructions. Briefly, cells were seeded in 96-well black plates (at the same densities indicated at the MTT viability assessment) with clear bottom and were left for 24 and 48 h incubation with the bioactive compounds. Antimycin-A (50 μM) was used as a positive control. After overnight incubation, the medium was aspirated, and cells were incubated for 10 min at 37 °C in 0.2 mL of measurement buffer containing 5 μM MitoSOX™ Red. Then, the cells were washed twice with PBS. MitoSOX™ fluorescence was measured at 510 nm excitation and 580 nm emission wavelengths on an Infinite M200PRO multimode microplate reader (Tecan Group Ltd., Männedorf, Switzerland). MitoSOX™ fluorescence was adjusted based on total protein content as determined by the Bradford assay [40] measured at an absorbance of 595 nm. The results were expressed as the percentage of adjusted values compared to control.

### 2.4. Measurement of Caspase Activity

The activity of caspase-3 enzymes was measured in cell lysates by a colorimetric assay kit (CASP-3-C, SIGMA-ALDRICH, Saint Louis, MO, USA), that is based on the hydrolysis of the compound acetyl-Asp-Glu-Val-Asp p-nitroanilide (Ac-DEVD-pNA) (A 2559, Sigma-Aldrich, Saint Louis, MO, USA) by caspase-3. The reaction releases the p-nitroaniline moiety (p-NA) that absorbs at 405 nm. Cells were pre-incubated with 5 μΜ doxorubicin hydrochloride (DOX) (Sigma–Aldrich, Deisenhofen, Germany) for 3 h for caspase cascade initiation [41,42]. Samples (10 μL) were tested both with and without the caspase-3 inhibitor acetyl-Asp-Glu-Val-Asp-al (Ac-DEVD-CHO, 20 μΜ final concentration), in a total reaction volume of 100 μL in 96-well plates. The substrate Ac-DEVD-pNA concentration was 200 μM and the assay was performed at 37 °C for 90 min. The caspase-3 specific activity was expressed as the percentage of μmol of p-nitroaniline released per min per μg of total protein values compared to control.

### 2.5. HeLa Cell Total Protein Extraction

For total protein extraction, cells were lysed in assay buffer (20 mM HEPES, pH 7.4, 2 mM EDTA, 0.1% CHAPS, 5 mM DTT) containing protease inhibitors (11873580001; Roche Diagnostics; Mannheim, Germany). Protein concentration was determined by the Bradford assay [40]. Briefly, the medium was removed from cells cultured in six-well plates and cells were washed with PBS. Then, 50 μL of assay buffer was added in each well and cells were left for 20 min in −20 °C. After incubation, lysed cells were centrifuged at 15,000× *g* for 15 min at 4 °C and the supernatants were transferred to Eppendorf tubes. The cytoplasmic proteins were maintained at −80 °C until use.

### 2.6. Creation of Membrane-Engineered Cells and Bioensor Fabrication (Vero-SOD)

Membrane-engineered mammalian cells were created by the electroinsertion of the enzyme superoxide dismutase (SOD) into the membrane of Vero cell fibroblasts following the protocol of Moschopoulou et al. [43]. Initially, cells at a density of 3 × 10^6^ mL^−1^ were centrifuged at 1000 rpm for 2 min and the pellet was resuspended in PBS (pH 7.4). Afterwards, cells were incubated with 1500 U·mL^−1^ CuZnSOD (EC1.15.1.1) for 20 min at 4 °C and the mixture was transferred to electroporator (Eppendorf Eporator, Eppendorf AG, Germany) cuvettes. Electroinsertion was performed by applying four pulses of an electric field at 1800 V·cm^−1^. Then, cells were centrifuged at 1000 rpm for 2 min and resuspended in cell culture medium. Finally, the sensors were fabricated by mixing 1 volume of Vero-SOD cells with 2 volumes of 4% (w/v) sodium alginate solution and was added dropwise with the use of a 22G syringe in 0.8 M CaCl_2_. Cells were immobilized in calcium alginate, forming beads containing 75 × 10^3^ cells per bead with an approximate diameter of 2 mm. As already reported [39,43], the membrane potential of membrane-engineered Vero cell fibroblasts is affected by the interactions of electroinserted SOD molecules and superoxide anions, producing measurable changes in the membrane potential.

### 2.7. Biosensor Setup for Recording Superoxide Concentration and Data Processing

**Figure 1:**
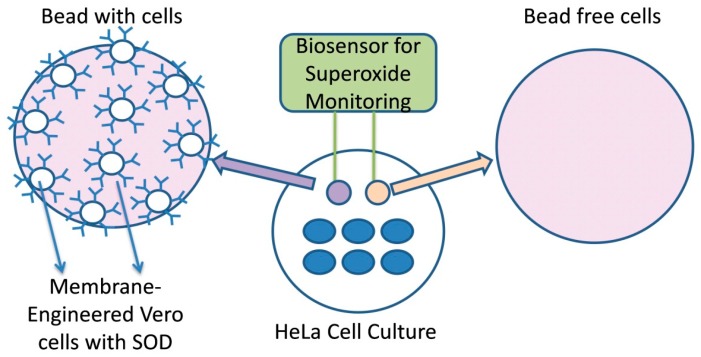
Cell-based biosensor system configuration.

For recording the signal and processing of data, the PMD-1608FSA/D card (Measurement Computing, Norton, MA, USA) recording device and the software InstaCal (Measurement Computing) were used, respectively. A two-electrode system (working and reference) was connected to the device. These silver electrodes were electrochemically coated with an AgCl layer. A cell-bearing bead was attached to the working electrode while a cell-free bead was connected to the reference electrode. For each assay, both beads (sensor system, Figure 1) were immersed into the well containing adherent cells [39] and the response of each biosensor potential was achieved within 100 s after its sinking into the culture medium. The biosensor was calibrated with known superoxide concentration produced by the oxidation of xanthine by the xanthine oxidase. The range of xanthine concentration was from 1 pM to 10 nM and the xanthine oxidase was 100 mU/mL [43]. Each response was expressed as the average of the cellular membrane potential of each assay, which has been calibrated to correspond to relative changes in superoxide concentration.

### 2.8. Statistical Analysis

Each experiment was repeated independently three times for each treatment with n = 5. Significance testing in comparisons was based on Student’s *t*-tests for pairs. *p*-values < 0.05 were considered to be statistically significant.

## 3. Results

### 3.1. Assessment of the Effects of the Chemotherapeutic Agent 5-Fluorouracil (5-FU) on HeLa Cell Viability

**Figure 2:**
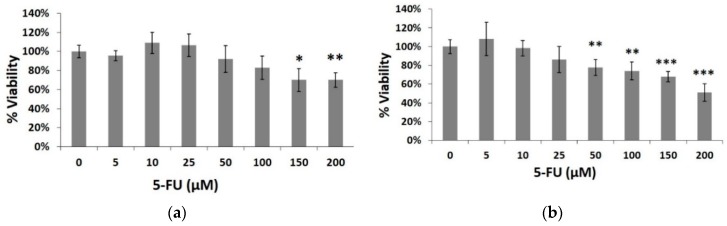
Percentage of HeLa cell viable cells after treatment with 5-fluorouracil (5-FU) at concentrations 5, 10, 25, 50, 100, 150 and 200 μΜ for (**a**) 24 h and (**b**) 48 h. Average results from replicate experiments ± SD (*n* = 3). * *p* < 0.05, ** *p* < 0.01, *** *p* < 0.001, significantly different from the control.

In order to study the 5-fluorouracil-induced cytotoxicity, HeLa cells were initially exposed to various concentrations of the cytostatic agent for 24 and 48 h. Doses up to 200 μΜ were used causing 30% and 49% inhibition of cell growth at 24 (Figure 2a) and 48 h (Figure 2b), respectively (at the highest dose of 200 μΜ).

### 3.2. Increased Mitochondrial Superoxide Production in HeLa Cells is Observed after 24 h Treatment with 5-FU

**Figure 3:**
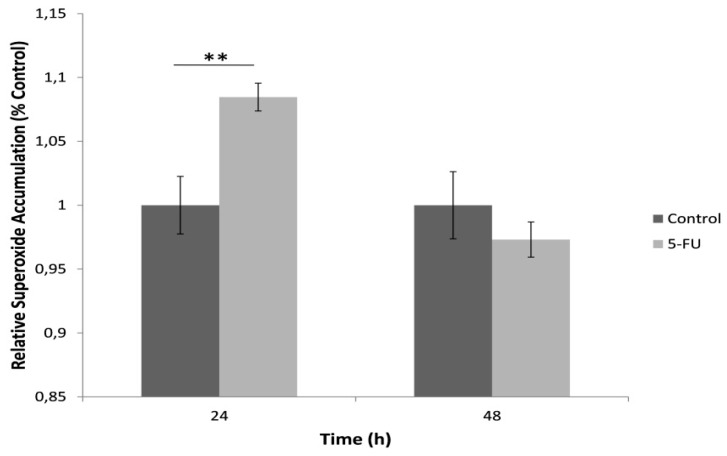
Mitochondrial superoxide levels in HeLa cells after treatment with 5-FU (150 μΜ) for 24 and 48 h, assessed as MitoSOX™ Red fluorescence intensity normalized to total protein content and control (no treatment with 5-FU). Average results from replicate experiments ± SD (*n* = 3). ** *p* < 0.01, significantly different from the control.

For the determination of mitochondrial superoxide production, cells were loaded with the fluorescent probe MitoSOX™ Red after exposure with a standard lethal 5-FU concentration (150 μΜ) for 24 h and 48 h (Figure 3). Our results indicated an increase in the mitochondrial superoxide levels in comparison with the control after 24 h cell exposure with 5-FU whereas a 48 h incubation led to a decrease in superoxide accumulation, possibly associated with cell loss due to 5-FU toxicity.

### 3.3. Caspase-3 Activation after 48 h Treatment with 5-FU

**Figure 4:**
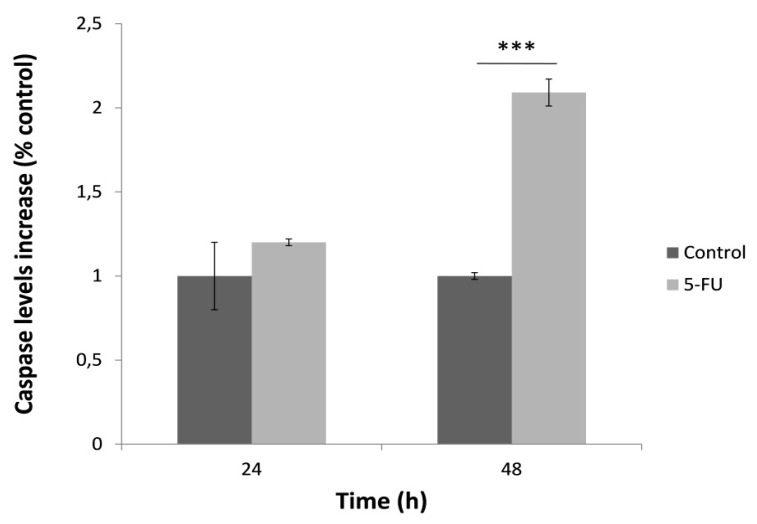
Caspase-3 specific activity in HeLa cells after treatment with 5-FU (150 μΜ) for 24 h and 48 h, normalized to control (no treatment with 5-FU). Average results from replicate experiments ± SD (*n* = 3). *** *p* < 0.001, significantly different from the control.

In order to determine the effect of 5-FU on the activation of caspase-3, HeLa cells were once again treated with 5-FU for 24 and 48 h and then the caspase specific activity was measured (Figure 4). HeLa cells treated for 24 h presented no significant caspase activity. On the contrary, caspase activation was remarkably increased at 48 h after treatment with 5-FU. Similar to mitochondrial superoxide levels, this observation was possibly associated with increased cell death after the 48-h treatment with 5-FU.

### 3.4. Superoxide Accumulation in Cell Culture Determined by the Bioelectric Cell-Based Biosensor

**Figure 5:**
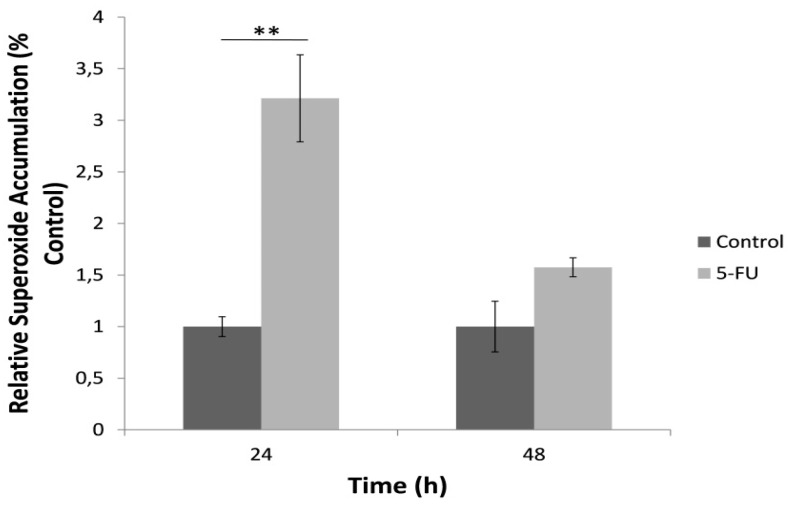
Relative superoxide accumulation in the culture medium during the 24 and 48 h incubation of HeLa cells with 5-FU, as determined with the cell-based superoxide biosensor, normalized to control (no treatment with 5-FU). Average results from replicate experiments ± SD (*n* = 3). ** *p* < 0.01, significantly different from the control.

A significantly increased superoxide concentration determined by the bioelectric biosensor-based assay was observed following the 24 h exposure to 5-FU compared to the control (Figure 5). On the other hand, we observed a significant reduction of superoxide levels after the 48 h treatment with the anticancer agent. The results of superoxide determination in the culture medium with the biosensor assay were very highly correlated with the respective results of the MitoSOX™ Red mitochondrial superoxide assay, though positively for the 24 h treatment (r^2^ = 0.99) and negatively for the 48 h treatment (r^2^ = −0.99).

## 4. Discussion

In this study, we demonstrate the successful implementation of a cell-based biosensor setup for the direct and rapid detection of superoxide released from cervical cancer cell culture treated with 5-FU, one of the most widely applied chemotherapeutic compounds in clinical therapy. Superoxide is considered a particularly significant ROS with an extremely short half-life; thus real-time level monitoring is an important yet complex issue. The in vitro determination of reactive oxygen species superoxide is generally assessed by spectrophotometric, analytical, electron spin resonance and electrochemical biosensor approaches [44]. Biosensors could provide reliable methods for the detection of relative superoxide levels in cell cultures without the need for extensive chemical and physical cell culture treatments.

The determination of dynamic radical changes in cellular microenvironment with non-invasive approaches is an essential mean for assessing the effectiveness of anticancer agents on cancer treatment [45,46,47,48]. As mentioned above, 5-FU’s chemotherapeutic properties are attributed to thymidylate synthase (TS) activity inhibition, to mitochondrial ROS generation and the caspases cascade activation [49]. Our findings demonstrate that 5-FU differentially affects superoxide production and caspase-3 activation when applied in cytotoxic concentrations against HeLa cells. MTT assay was used to evaluate the antiproliferative effect of 5-FU at 24 h and 48 h treatments. We spectrophotometrically determined elevated mitochondrial superoxide production after the 24-h treatment but no changes in caspase-3 activity. On the contrary, mitochondrial superoxide release was decreased to control levels whereas caspase-3 activity was significantly upregulated. These trends are in accordance with the results obtained from the superoxide biosensor as 5-FU significantly upraised superoxide production after the 24 h treatment, followed by a downregulation to control levels after 48-h incubation, possibly also associated with increased number of cells undergoing apoptosis.

The correlation between the response of the superoxide bioelectric biosensor and the superoxide concentration is indirectly linked to changes caused in the cell membrane potential of cells which have been membrane-engineered with SOD moieties, i.e., SOD units which have been electroinserted in the cell membrane. This technology is known as molecular identification through membrane engineering. This is a generic methodology of artificially inserting tens of thousands of receptor molecules on the cell surface, thus rendering the cell a selective responder against analytes binding to the inserted receptors. Receptor molecules can vary from antibodies to enzymes to polysaccharides [50,51,52,53,54]. It has been previously proven [55] that this principle is associated, in a unique way, with certain changes in the cellular electric properties (in particular, cell membrane hyperpolarization) as a consequence of the interaction of the analytes under determination with the electroinserted molecules and therefore changes in the cellular structure.

It must be emphasized that cell membrane hyperpolarization is the dominant change in the engineered cell membrane electric properties following a mechanical distortion of the membrane (according to the novel assay principle described in the method) as also expected by the concurrent change in the actin cytoskeleton structure, in particular the actin cytoskeleton network adjunct to the sites of the interaction, including the circumferential actin belt and changes in the propagation of electric signals along actin filaments [56]. On the contrary, the main change in the cell membrane electric properties of normal (i.e., non-membrane engineered) cells is depolarization, as frequently described in prior approaches [57,58,59].

It has been previously proven that superoxide dismutation triggered changes to the membrane potential of fibroblast cells membrane-engineered with SOD [43]. In order to measure the aforementioned changes in the engineered cell membrane potential, the superoxide biosensor used in the present study was designed according to the principle of the bioelectric recognition assay (BERA) [57]. This methodology has been often applied to detect cellular interactions with bioactive compounds via the determination of electrical conductivity generated adjacent to a cell cluster as a means of indirect measurement of the relative changes in the cell membrane potential. This means that the AgCl electrode system applied is able to measure conductivity alterations of the extracellular microenvironment reflecting the bioelectric profiling of cell responses to various treatments [60]. It has been previously demonstrated that by using this superoxide biosensor system it is possible to rapidly measure superoxide concentrations as low as 1 pM [43] and also to correlate the biosensor response with currently available conventional methods for superoxide determination [43,50,61].

Mitochondrial superoxide is produced when electrons released from the electron transfer system in the inner membrane of mitochondria are captured by molecular oxygen and become superoxide [62]. It has been reported that extracellular superoxide is closely linked to mitochondrial superoxide production [63]. Our results support this statement as high mitochondrial superoxide levels, measured with mitochondria-targeted MitoSOX™ Red fluorogenic dye are accompanied by high extracellular accumulation of the anion. However, superoxide concentration data recorded with the cell-based biosensor should be considered with caution compared with MitoSOX™ results, since the biosensor assayed superoxide eluted in the culture medium, not its actual intracellular concentration. That said, it is worth elaborating on the observation that, although the results obtained with the bioelectric biosensor for the 24 h treatment were very highly and positively correlated with the MitoSOX™ assay, a negative yet high correlation was established for the 48-h treatment. This could be possibly explained by the reduced number of live HeLa cells after their prolonged exposure to 5-FU. As already hypothesized in Section 3, this would result in a lower level of superoxide accumulation in the mitochondria (since fewer cells will be metabolically active) and at the same time in a higher level of superoxide released in the culture media (compared to control) due to cell membrane leakage from dead cells. In this respect, the reliability of the biosensor-based assay in accessing the ROS-mediated toxicity of 5-FU is considerably higher than the MitoSOX™ assay, since it allows for a more realistic and accurate estimation of the level of oxidative stress related to and/or resulting from the anticancer drug effect.

## 5. Conclusions

Intracellular ROS are increasingly recognized as critical determinants of cellular signaling and can be used as markers of 5-FU-mediated anti-tumor effects. Biosensors are widely used for the assessment of physiological and pathological applications since they are non-invasive methods for the detection of crucial biomarkers of cancer cell cytotoxicity without the need for extensive sample pretreatment. In this study, we present a cell-based biosensor tool suitable for cell culture superoxide monitoring without the need for cell transfer and staining, which could affect cellular responses due to extrinsic chemical and mechanical stress. This biosensor is applied for the estimation of total superoxide levels released from the cancer cells into the culture medium after treatment with the chemotherapeutic agent. Our results are in agreement with the findings of the biochemical assays for the assessment of mitochondrial superoxide and caspase-3 activities. Further validation is required of the feasibility of our biosensor-based approach for monitoring alterations in the in vitro redox status of cancer cells treated with anticancer agents, e.g., by testing more and different cancer cell lines crossed with chemotherapeutical drug combinations. In a longer perspective, the availability of a reliable, high throughput and rapid system for superoxide determination in cancer cells will allow for the accurate assessment of chemoresistance in cervical and other cancer cells, at least as far as its association with redox balance is concerned [38,64,65,66].
